# Myasthenia gravis and community-acquired pneumonia: therapeutic challenges

**DOI:** 10.3389/fphar.2025.1695526

**Published:** 2025-11-13

**Authors:** Xueying Chen, Jianbo Ding, Jiali Zhang, Haibin Dai, Lingyan Yu

**Affiliations:** 1 Department of Pharmacy, The Second Affiliated Hospital, School of Medicine, Zhejiang University, Hangzhou, China; 2 Department of Emergency Medicine, The Second Affiliated Hospital, School of Medicine, Zhejiang University, Hangzhou, China

**Keywords:** myasthenia gravis, community-acquired pneumonia, neuromuscular junction disorders, immunocompromised, pneumonia, drug‒drug interactions

## Abstract

Myasthenia gravis (MG) patients are highly susceptible to community-acquired pneumonia (CAP) due to the need for immunosuppressive therapies and aspiration risks, with CAP representing the leading infectious cause of mortality in this population. The intersection of MG and CAP poses unique challenges for the management of anti-infective agents and immunosuppressants. There is currently no systematic literature review addressing these issues, as previous reviews have been limited to one of these aspects. This review synthesizes evidence on the pharmacotherapeutic challenges associated with MG-CAP comorbidity, focusing on three key areas: avoiding antibiotics that exacerbate neuromuscular junction symptoms, minimizing drug interactions, and managing infection-adjusted immunosuppressants. Through a comprehensive synthesis of literature, we provide recommendations for optimizing antibiotic selection and immunosuppressants while tailoring immunosuppressive strategies according to CAP severity grading. This facilitates optimal management of both MG and infection control, highlighting the need for dynamic, patient-centered approaches. This clinical decision-making tool serves as a practical reference for physicians in the absence of established guidelines or expert consensus for managing this complex patient population.

## 1 Background

Myasthenia gravis (MG) is an autoimmune disorder characterized by impaired neuromuscular junctions, leading to fluctuating muscle weakness in ocular, limb, bulbar, and respiratory regions (Gilhus et al., 2019; Punga et al., 2022). In recent decades, the global prevalence of MG has increased substantially, with mortality rates disproportionately increasing among Chinese adolescent males and elderly individuals, reflecting persistent challenges in therapeutic management (Jiang et al., 2023; Zhang et al., 2023). Patients with MG are at increased risk of pneumonia (16%–41.18%), which is attributable to chronic immunosuppression and susceptibility to aspiration (Sipilä et al., 2019; Prior et al., 2018; Kassardjian et al., 2020). Pneumonia represents a major contributor to clinical deterioration and mortality in this population (Wang et al., 2024; Su et al., 2022a). Community-acquired pneumonia (CAP), a common infectious disease, is a leading cause of global morbidity and mortality, particularly in vulnerable groups such as individuals with comorbidities such as MG (Vaughn et al., 2024; Berkel et al., 2016).

In patients with MG, antibiotic selection may have a profound effect on the disease trajectory. Certain antibiotic classes, such as aminoglycosides and fluoroquinolones, are strongly associated with the induction of myasthenic crises through interference with neuromuscular transmission (Berkel et al., 2016). Furthermore, disease exacerbation and immunosuppressive regimens may heighten susceptibility to CAP or exacerbate its severity, likely through respiratory muscle dysfunction and systemic immunosuppression (Gilhus, 2023). Optimal management of infections and acute exacerbations in MG is paramount. Timely initiation of narrow-spectrum antibiotics guided by microbiological evidence, alongside judicious titration of immunosuppressive agents, may mitigate the risk of severe complications such as respiratory failure and improve clinical outcomes, particularly in CAP-related contexts.

Empiric antibiotics are frequently initiated in emergency or outpatient settings, increasing the risk of inadvertently prescribing agents that precipitate myasthenic crisis (MC) (Berkel et al., 2016). When infection drives clinical deterioration in MG patients, two critical questions arise: How can antibiotic selection be optimized to balance microbial coverage and safety? Should immunosuppressive therapy be maintained or temporarily withheld on the basis of infection severity? Despite the clinical urgency, no systematic review has holistically addressed these interconnected dilemmas, as prior studies have focused narrowly on isolated aspects of MG management (Berkel et al., 2016; Gilhus, 2023; Muppidi et al., 2020).

In conclusion, the management of patients with comorbid MG and CAP poses distinct therapeutic challenges, particularly in optimizing the balance between anti-infective agents and immunosuppressive protocols. By synthesizing and appraising contemporary evidence on pharmacotherapeutic strategies for this population, this review aims to establish evidence-informed recommendations for clinical decision-making and improve patient prognosis through targeted pharmacotherapeutic strategies, including the avoidance of antibiotics implicated in neuromuscular junction dysfunction, the mitigation of pharmacokinetic interactions, and the adaptation of immunosuppressant (IS) regimens according to infection status. The systematic implementation of these interventions has the potential to substantially alleviate the compound disease burden imposed on healthcare systems.

## 2 Evidence grading methodology

The evidence levels referenced in this article are classified using the Oxford Centre for Evidence-Based Medicine (CEBM) 2011 Levels of Evidence (Nuffield Department of Primary Care Health Sciences, 2025), and detailed information is provided in Supplementary Table S2. This system categorizes study designs based on their methodological rigor and potential for bias, with Level 1 representing the strongest evidence and Level 5 the weakest. This grading applies specifically to the design of the original studies being cited, rather than to this review itself. The purpose of this classification is to provide readers with a clear and immediate understanding of the strength of the evidence supporting each clinical statement. It enables healthcare professionals to distinguish between recommendations grounded in robust research and those derived from less definitive sources, thereby supporting more informed clinical decision-making.

## 3 Myasthenia gravis

Myasthenia gravis (MG) is an autoimmune disorder characterized by fatigable muscle weakness. Effective management of this disease requires a thorough understanding of its clinical subtypes, the limitations of existing therapies, and a critical challenge in treatment: the inherent trade-off between immunosuppression and the increased risk of infection, which is particularly relevant in the context of concurrent community-acquired pneumonia (CAP) (Prior et al., 2018). This section will review the disease classification, conventional and novel treatment strategies, and their associated risks.

### 3.1 Disease classification and clinical features

MG is clinically stratified into ocular MG and generalized subtypes, with the latter constituting approximately 85% of cases and potentially progressing to respiratory muscle weakness or life-threatening respiratory failure in severe forms (Paz and Barrantes, 2019). Pathogenic autoantibodies in MG predominantly target components of the neuromuscular junction (NMJ), thereby impairing synaptic transmission. The major subtypes of autoimmune MG are classified on the basis of the specific antigenic targets of these autoantibodies. Approximately 85% of patients harbor autoantibodies against the nicotinic acetylcholine receptor (AChR), while less frequent targets include muscle-specific kinase (MuSK) and lipoprotein receptor-related protein 4 (LRP4) (Fichtner et al., 2020). Notably, approximately 15% of patients may develop MC, a life-threatening neurological emergency manifesting as acute respiratory failure (Gilhus et al., 2019).

### 3.2 Current treatment strategies and challenges

The traditional treatment for MG encompasses cholinesterase inhibitors, glucocorticoids (GCs), IS, intravenous immunoglobulins (IVIg), plasma exchange (PE), and thymectomy, which collectively achieve symptomatic control in the majority of patients (Gilhus et al., 2019). However, immunosuppressive regimens are associated with substantial long-term adverse drug reactions (ADRs), particularly during chronic administration, which complicates clinical management, especially regarding infection risks (as detailed in Supplementary Table S2) (Level 5) (Malpica and Moll, 2020). Approximately 15% of patients exhibit limited or no response to conventional treatments, underscoring the need for novel therapeutic strategies. The emergence of molecular therapies, including monoclonal antibodies, B-cell-depleting agents, and chimeric antigen receptor T-cell-based therapies, has the potential to revolutionize the MG treatment landscape (Iorio, 2024).

### 3.3 Individual therapies: mechanisms, limitations, and infection risks

#### 3.3.1 Symptomatic treatment and its limitations in crisis

Symptomatic treatment with acetylcholinesterase inhibitors enhances neuromuscular transmission by increasing synaptic acetylcholine levels. Although this intervention enhances respiratory muscle strength, dose-dependent side effects such as excessive airway secretions may impair ventilation and increase infection risk, especially in patients with preexisting respiratory weakness (Level 5) (Gilhus, 2023). Current guidelines therefore recommend withholding these agents during ventilator-dependent phases of MC (Wiendl et al., 2023).

#### 3.3.2 Immunosuppressive therapies and novel agents

Although IS and thymectomy improve generalized muscle weakness, they have no direct therapeutic effect on the respiratory musculature. Paradoxically, the management of respiratory insufficiency frequently necessitates escalation of immunosuppressive therapy to halt disease progression. Novel immunomodulators, such as complement inhibitors and neonatal Fc receptor (FcRn) blockers, exhibit high efficacy and favorable safety profiles in antibody-positive MG patients refractory to conventional therapies. However, their elevated cost and limited regulatory approval for severe respiratory subtypes limit their widespread clinical application (Schett et al., 2024; Howard et al., 2021).

#### 3.3.3 Rapid-acting interventions and bridging therapies

Therapeutic PE and IVIg serve as rapid-acting immunomodulatory interventions in MG, with clinical effects manifesting within days, and the benefits are only short term (weeks), which are mainly used in acute exacerbations and as a “bridging” measure to slower-acting immunotherapies (such as azathioprine [AZA] or mycophenolate mofetil [MMF]), and may be considered before thymectomy or other surgical procedures.

## 4 Summary of infection risk

Except for IVIg, all current MG therapies increase susceptibility to diverse microbial infections (viral, bacterial, fungal, parasitic), and ISs are particularly implicated in this risk profile (Level 5) (Chiu and Chen, 2020). Although immunosuppression effectively controls MG progression, it concurrently reshapes infection risk profiles, particularly manifesting as altered pathogen distribution and atypical therapeutic responses in CAP, a complexity that demands systematic analysis in subsequent management strategies.

## 5 Community-acquired pneumonia in immunocompromised adults

CAP is caused by an infection of the lung parenchyma that occurs outside of a hospital setting and results from pathogens infecting the lower respiratory tract. The ensuing infection and inflammatory response lead to respiratory (e.g., cough, dyspnea) and systemic (e.g., fever) symptoms and may result in sepsis, acute respiratory distress syndrome, and death. CAP is one of the leading causes of death in children, elderly individuals, and immunocompromised individuals (Level 1) (Vaughn et al., 2024; Tsoumani et al., 2023). Despite its clinical significance, only 38% of hospitalized CAP patients achieve microbiological confirmation. Viral etiologies account for up to 40% of identified cases, whereas *Streptococcus pneumoniae*, the predominant bacterial pathogen, is detected in only 15% of patients (Vaughn et al., 2024). CAP remains an underrecognized yet prevalent clinical entity, particularly in immunocompromised hosts. In this population, classical CAP signs are often attenuated, and patients may initially present as clinically stable, only to experience rapid clinical deterioration—a phenomenon strongly associated with delayed diagnosis (Aliberti et al., 2021; Ramirez et al., 2020). An estimated 3% of the United States (US) adult population is immunocompromised, and 20%–30% of CAP-related hospitalizations occur in this vulnerable subgroup (Level 3) (Di Pasquale et al., 2019).

Immunocompromised patients exhibit susceptibility to core respiratory pathogens (e.g., *Streptococcus pneumoniae*, respiratory viruses) that commonly cause CAP in immunocompetent individuals, albeit with heightened severity (Level 5) (Aliberti et al., 2021). In addition to these pathogens, clinicians must consider opportunistic and drug-resistant organisms in immunocompromised hosts, including carbapenem-resistant *Enterobacteriaceae*, *Mycobacterium* species, cytomegaloviru*s*, *Pneumocystis jirovecii*, *Cryptococcus*, *Nocardia, etc.* Different types of immunocompromising conditions are predisposed to different types of etiologic agents (Ramirez et al., 2020; Wu et al., 2023). Notably, in MG patients, bacterial pathogens account for 90.32% of pneumonia cases, with carbapenem-resistant strains identified in 42.86% of isolates. Nonfermenting gram-negative bacilli (e.g., *Pseudomonas aeruginosa*) are the most common microorganisms (Su et al., 2022a).

## 6 Community-acquired pneumonia in patients with myasthenia gravis

MG is characterized by three cardinal features: skeletal muscle weakness, autoimmune-driven pathology, and dependence on chronic immunosuppressive therapy. These factors can contribute to susceptibility to respiratory tract infections, increase their severity and complication risks, and affect the management and prevention of respiratory infections (Gilhus, 2023). Approximately 70% of patients with generalized MG exhibit bulbar muscle weakness and dysphagia, a significant risk factor for aspiration (Su et al., 2022a). A case‒control study revealed a 3.17-fold elevated pneumonia risk in MG patients with dysphagia (95% confidence interval [CI]: 2.07–4.87), increasing to 11.56-fold (95% CI: 3.36–39.77) in those with documented aspiration (Level 4) (Martino et al., 2005). MG pathogenesis is driven by a dysregulated immunopathological cascade encompassing T-cell dysfunction, B-cell hyperactivation, complement system overactivation, and thymic abnormalities. These perturbations collectively impair pathogen clearance and immune surveillance, increasing susceptibility to infection (Zhang et al., 2021; Tackenberg et al., 2018). GCs and therapies that inhibit B-cell or T-cell responses are the most commonly used therapies, leading to impaired immune function and infection with various respiratory pathogens (Table 1) and increasing the risk of respiratory infections by 20%–50% in MG patients (Di Pasquale et al., 2019; Gilhus et al., 2018).

**TABLE 1:** DDI between anti-infective agents and IS.

Drug interaction combinations	Risk rating	Adverse effects or impacts on concentration	Monitoring and management	Comment
GCs + Triazoles	C	GCs^a^↑	Steroid-related adverse effects	prednisone + itraconazole/fluconazole: B
GCs + Amphotericin B	C	The hypokalemic effect of Amphotericin B ↑	Cardiac function and serum electrolytes (especially potassium)	—
GCs + Rifamycins	C	GCs^a^ ↓	Effects of steroid	Rifampin + MP/DXM: D
GCs + isoniazid	C	isoniazid^a^ ↓	Effects of isoniazid	—
GCs + CIarithromycin	C	GCs^a^ ↑	Steroid-related adverse effects	Avoid this combination for patients using DXM to treat life-threatening conditions
GCs + Quinolones	C	The risk of tendonitis and tendon rupture ↑	new onset of tendon or joint pain	The risk of tendonitis and tendon rupture may be further increased in older patients and organ transplant recipients
MP/DXM + NMV/r	C	GCs^a^ ↑	Steroid-related adverse effects	—
DXM + caspofungin	D	caspofungin^a^↓	Effects and considering an increased caspofungin dose of 70 mg daily in adults	Considering 70 mg/m^2^, up to a maximum of 70 mg daily in pediatric patients
CTX + Rifampin/(NMV/r)	C	the active metabolite(s)^a^ of CTX ↑	CTX toxicities (e.g., mucositis, neutropenia)	—
CTX + Fluconazole/Itraconazole	C	CTX^a^ ↑	Serum bilirubin and serum creatinine	—
AZA/CTX/MTX + Linezolid	C	The myelosuppressive effect ↑	Complete blood counts weekly	—
AZA + SMZ-TMP	C	The myelosuppressive effect ↑	Immune function and hematologic status CIosely	—
AZA + Ribavirin	D	The potentially myelotoxic methylated metabolites of AZA ↑	Considering alternative agent(s) when possible	—
MTX + Sulfonamide Antibiotics	D	The myelosuppressive effect ↑	Immune function and hematologic status closely	Avoiding concomitant use of MTX and therapeutic doses of sulfonamides
MTX/CTX + Penicillins/Cephalosporins/TetracyCIine	C	MTX^a^ ↑ The hypokalemic effect of Antineoplastic Agents↑(only for piperacillin)	Toxic effects of MTX, including neutropenia and hypokalemia	CTX only interacts with piperacillin
MTX + Voriconazole	C	The photosensitizing effect of Voriconazole ↑	Photosensitivity reactions and cheilitis	When voriconazole is combined with MTX injection
MTX/CTX + Amphotericin B	C	The adverse/toxic effects of Amphotericin B ↑	Possible increases in renal toxicity, bronchospasm, and hypotension	—
MTX + Pyrimethamine	C	The adverse/toxic effects of MTX ↑	Increased hematologic toxicities and folate deficiency	If signs of folate deficiency develop, pyrimethamine should be discontinued. Folinic acid should be administered until normal hematopoiesis is restored
MTX + Levofloxacin/Ciprofloxacin	C	MTX^a^ ↑	MTX toxicities or delayed MTX elimination	—
MMF + Antibiotics	C	Mycophenolate^a^ ↓	Concentrations and effectiveness of mycophenolate	MMF + rifampin: D, Not recommended to be given with rifampin
MMF + Isavuconazole	C	Mycophenolate^a^ ↑	Evidence of increased mycophenolate clinical effects	—
MMF + Ganciclovir/Valganciclovir	C	The risk for leukopenia or neutropenia ↑	Ganciclovir, valganciclovir and mycophenolate toxicities	—
TAC + Rifamycins	C	TAC^a^ ↓	Decreased concentrations and effects, adjust doses as needed	Rifampin + TAC: D, larger dosages of TAC may be needed
TAC + Triazoles	D	TAC^a^ ↑	TAC concentrations CIosely beginning within 1–3 days of concomitant use and adjust dose as necessary	Isavuconazole + TAC: C; Reduce TAC dose to approximately one-third of the original dose when starting concurrent voriconazole and Posaconazole
TAC + NMV/r	D	TAC^a^↑	Increased TAC concentrations and toxicities. NIH COVID-19 treatment guidelines recommend holding TAC during NMV/r treatment and for at least 2–3 days after completion	Use of alternative COVID-19 therapy is recommended by the American Society of Transplantation (AST)
TAC + CIarithromycin	D	TAC^a^↑	TAC dose reductions and/or prolongation of the dosing interval will likely be required	
TAC + Remdesivir/Azithromycin/Ciprofloxacin	C	TAC^a^↑	Increased TAC concentrations and toxicities	Adjust doses as needed
TAC + Amphotericin B/Cidofovir	C	The nephrotoxic effect of TAC ↑	Renal function	—
TAC + Levofloxacin	C	TAC^a^↑ the QTc-prolonging effect of Levofloxacin↑	Signs and Symptoms of excessive QTc interval prolongation and arrhythmia; TAC concentrations and toxicities	—
CsA + Triazoles	D	CsA^a^ ↑	CsA concentrations and serum creatinine	CsA + Fluconazole/Isavuconazole: C; Reduce CsA dose by 50%–80% when starting concurrent voriconazole, and 25%–50% when starting concurrent itraconazole or posaconazole
CsA + amphotericin B/SMZ-TMP/GanciCIovir-ValganciCIovir/Ciprofloxacin	C	Nephrotoxic effects of CsA ↑	Renal dysfunction, an alternative drug or an adjustment to CsA dose as needed	Newer amphotericin formulations (such as lipid complex, liposomal, cholesteryl sulfate complex) may be safer options
CsA + Rifamycins/isoniazid/Sulfadiazine	C	CsA^a^ ↓	CsA serum concentrations and effects	Rifampin + CsA: D, larger dosages of CsA may be needed
CsA + pyrazinamide	c	The myopathic (rhabdomyolysis) effect of CsA ↑	CsA concentrations and muscle toxicities	—
CsA + Caspofungin	D	CsA^a^↑ Caspofungin^a^↑	Weigh the potential benefits of caspofungin therapy against a possible elevated risk of hepatotoxicity in patients receiving CsA and monitor liver function	Re-evaluate the potential risks and benefits of treatment in patients with abnormal liver function
CsA + Remdesivir	C	CsA^a^ ↑ Remdesivir^a^ ↑	CsA concentrations and toxicities	CsA dose adjustments may be needed
CsA + Imipenem and Cilastatin	C	The neurotoxic effect of CsA ↑	Signs and symptoms of neurotoxicity	—
CsA + Linezolid	C	Linezolid^a^ ↑	Linezolid toxicities	—
CsA + NMV/r	D	CsA^a^ ↑	NIH COVID-19 treatment guidelines recommend a CsA dose adjustment. Limited evidence supports a CsA dose reduction of 80%. The timing of CsA dose increases after NMV/r completion should be guided by continued monitoring of CsA levels	Avoid nirmatrelvir/ritonavir in patients receiving CsA if CIose monitoring of CsA concentrations is not possible. Use of alternative COVID-19 therapy is recommended by AST.
CsA + Clarithromycin	D	CsA^a^ ↑	CsA dose reductions and/or prolongation of the dosing interval will likely be required	—
CsA + Azithromycin	C	CsA^a^ ↑	CsA concentrations and toxicities	—

Respiratory infections act as both triggers and amplifiers of MG exacerbations (Sansoni et al., 2023). Cohort data indicate that approximately one-third of MG relapses and severe exacerbations with respiratory dysfunction are caused by respiratory infections (Level 4) (Ramsaroop et al., 2023). A retrospective analysis of 86 MG patients revealed that early-onset MG (adjusted OR: 3.079, 95% CI 1.052–9.012) and respiratory infection (adjusted OR: 3.926, 95% CI 1.141–13.510) were independent risk factors for progression to MC, whereas IVIg treatment (adjusted OR: 0.253, 95% CI 0.087–0.732) before mechanical ventilation was a protective factor (Level 3) (Huang et al., 2021). In elderly MG patients, CAP frequently results in respiratory failure during the summer months, necessitating intensive care admission for ventilator support. The main symptom of an impending MC is progressive weakness of the respiratory and bulbar muscles. Early and immediate hospitalization with admission to an intensive care unit is necessary (Level 2) (Nelke et al., 2022).

The management of MG complicated by CAP necessitates confronting the inherent contradiction between immunosuppression and infection control. Immunosuppressive drugs, in combination or at high doses, alleviate MG symptoms but increase the risk of respiratory infections, particularly those associated with carbapenem-resistant pathogens and aspiration-related pneumonia. Clinical practice demands dynamic prioritization-maintaining baseline immunosuppression to prevent myasthenic crises while maintaining heightened alertness for infectious signs (treating even minor infections as emergencies). Early initiation of antibiotic regimens covering drug-resistant bacteria and opportunistic pathogens, guided by microbiological profiles, coupled with real-time adjustments to immunosuppressive intensity on the basis of inflammatory biomarkers (e.g., procalcitonin) and respiratory function monitoring, is critical. Crucially, even possibly minor infections necessitate aggressive intervention to avert clinical deterioration, underscoring the need for a preemptive, risk-stratified approach that harmonizes immunosuppression optimization with infection containment in this high-risk cohort.

## 7 Selection and use of antibiotics

### 7.1 General principles and high-risk antibiotic classes

Patients with MG complicated by CAP face increased susceptibility to diverse pathogens (bacterial, fungal, and viral) due to combined immunodeficiency from both the disease itself and chronic immunosuppressive therapies (Ramirez et al., 2023). Antibiotic selection requires meticulous consideration, as certain agents may impair neuromuscular transmission through presynaptic blockade of acetylcholine release or postsynaptic receptor antagonism, thereby exacerbating MG symptoms (Level 4–5) (Berkel et al., 2016; Sheikh et al., 2021). Concurrently, pharmacokinetic and pharmacodynamic interactions between ISs and antimicrobials critically influence therapeutic efficacy and safety. Optimal antimicrobial therapy in this context must integrate pathogen susceptibility profiles with rigorous evaluation of drug interactions affecting IS pharmacokinetics (e.g., cytochrome P450 modulation) and toxicity thresholds (Level 5) (Kaufman et al., 2004) (see Table 1) for a comprehensive interaction matrix derived from Lexicomp® (Lexicomp®, 2025).

International consensus guidelines strongly contraindicate aminoglycosides, macrolides, and fluoroquinolones in MG management because of their neuromuscular toxicity (Narayanaswami et al., 2021). To date, many MG exacerbations have been reported with systemic exposure to these antibiotics (Gummi et al., 2019; Harnett et al., 2009; Krenn et al., 2020; Pham et al., 2021). A retrospective cohort study of adult MG patients demonstrated that fluoroquinolones posed significantly greater 15-, 30-, and 90-day hospitalization risks than macrolides did (Level 3) (Pham et al., 2021). Among macrolides, azithromycin has been most frequently implicated in medication-triggered exacerbation. Aminoglycosides are contraindicated in MG patients because of their neuromuscular toxicity, particularly in critically ill patients with renal impairment or who are receiving concurrent neuromuscular blockers (Gummi et al., 2019). However, tobramycin may be a safer aminoglycoside option when it is administered at standard antibacterial concentrations (Level 4) (de Rosayro and Healy, 1978). Given the substantial risk of myasthenic crises associated with these antibiotic classes, avoidance is strongly recommended unless no alternatives exist (Marriott et al., 2023).

### 7.2 Recommended and alternative antibacterial agents

Tetracyclines, including doxycycline and minocycline, offer a viable alternative for CAP management in MG patients. While competitive AChR antagonism occurs at supratherapeutic doses (Friedrich et al., 2004),clinical studies confirm its safety within standard therapeutic ranges (Mihevc et al., 2021; Novitch et al., 2018) (Level 4) and suggest potential protective effects against neuromuscular toxicity in preclinical models (Davies et al., 2005). Importantly, tetracyclines demonstrate minimal pharmacokinetic interactions with the IS in MG, further confirming their role as first-line agents for CAP in suspected atypical pathogens (Level 4) (Berkel et al., 2016).

Although β-lactam antibiotics are generally considered safe for infections in MG (Level 5) (Sheikh et al., 2021), few reports of MG exacerbation exist. Recent studies document six cases of MG symptom worsening following amoxicillin therapy (Vacchiano et al., 2020) and two ampicillin-induced relapses confirmed by drug rechallenge (Argov et al., 1986). In contrast, cephalosporins demonstrate no significant transmission interference in experimental models, with no documented cases of MG exacerbation reported in the literature to date (Level 5) (Friedrich et al., 2004; Deng et al., 2005). Carbapenems are the empirical therapy of choice for severe pulmonary infections caused by unidentified or multidrug-resistant pathogens, particularly in immunocompromised hosts (Vaughn et al., 2024). To date, carbapenem-associated MG exacerbations have been documented only in isolated case reports (Level 4) (Margolin, 2004; O’Riordan et al., 1994). Given their overall safety profile and broad antimicrobial activity, β-lactams retain first-line status for MG-related infections (Level 5). Clinicians must nevertheless remain vigilant for pharmacokinetic interactions, including penicillin/cephalosporin-induced methotrexate (MTX) accumulation, piperacillin-exacerbated hypokalemia with antineoplastics, imipenem-cilastatin neurotoxicity in cyclosporine (CsA)-treated patients, and reduced mycophenolate bioavailability with multiple antibiotics (Crescioli et al., 2023; Zarychanski et al., 2006; Jafari et al., 2023). Proactive therapeutic drug monitoring and rapid clinical reassessment are therefore essential during treatment to detect early signs of MG deterioration or toxicity.

In the existing published studies, no MG exacerbations have been reported with vancomycin, linezolid, or sulfonamides (including trimethoprim-sulfamethoxazole [SMZ-TMP]). Polymyxins are reserved for carbapenem-resistant gram-negative infections but carry neurotoxic risks mediated by neuronal lipid interactions (Honore et al., 2013; Zu et al., 2021). The most severe manifestation is neuromuscular blockade, which presents as respiratory paralysis with apnea or myasthenia gravis-like syndromes and typically occurs at supratherapeutic drug levels or with concomitant neurotoxic agents. Despite these mechanistic risks, clinically significant events remain exceedingly rare in real-world practice (Level 5) (Spapen et al., 2011; Özkan et al., 2012). Concomitant administration of linezolid or SMZ-TMP with myelosuppressive agents (MTX, cyclophosphamide [CTX], AZA) potentiates hematologic toxicity, with MTX and SMZ-TMP combinations at therapeutic doses being strictly contraindicated owing to synergistic folate antagonism. Concurrent use of SMZ-TMP and CsA further exacerbates nephrotoxic risks through competitive impairment of tubular secretion. These interactions necessitate vigilant monitoring of complete blood counts and renal function parameters during therapy (Kaufman et al., 2004).

### 7.3 Antifungal agents in CAP management

Current evidence indicates that no MG exacerbations are associated with amphotericin B, caspofungin, or nonvoriconazole triazoles in fungal CAP management. However, voriconazole has rarely been implicated in MG deterioration (Level 4) (Azzam et al., 2013; Akcam et al., 2022). Triazole antifungals potently inhibit cytochrome P450 enzymes (CYP3A4/CYP2C19), significantly altering the pharmacokinetics of coadministered MG therapies (Level 4) (Brüggemann et al., 2022). Lexicomp® data (Lexicomp®, 2025) highlight critical interactions: CYP3A4 inhibition elevates corticosteroid and calcineurin inhibitor (CNI) exposure, whereas isavuconazole increases mycophenolate bioavailability. Therapeutic drug monitoring and dose titration are thus mandatory, and isavuconazole is superior to other triazole drugs in terms of efficacy and safety, making it more suitable for the treatment of pulmonary fungal infections in MG patients (Level 1) (Domingos et al., 2022; Ullmann et al., 2018). Concurrent administration of amphotericin B with IS amplifies amphotericin B-associated toxicities, notably hypokalemia and dose-dependent nephrotoxicity. However, lipid-based amphotericin formulations (e.g., liposomal amphotericin B) significantly reduce renal toxicity while maintaining antifungal efficacy, positioning them as preferred alternatives in immunocompromised populations (Level 2) (Qin et al., 2025). Caspofungin metabolism is accelerated by dexamethasone, necessitating dose escalation to 70 mg/day in adults (Level 2) (Sable et al., 2002). Although CsA‒caspofungin combinations theoretically increase hepatotoxicity, clinical studies in transplant recipients have revealed minimal hepatic injury, supporting cautious use with liver function monitoring (Level 4) (Christopeit et al., 2008; Saner et al., 2006).

### 7.4 Antiviral agents and special considerations for tuberculosis

MG exacerbations are associated with diverse viral infections, including varicella-zoster virus, cytomegalovirus, SARS-CoV-2, influenza, adenovirus, and respiratory syncytial virus, whereas certain antiviral therapies may independently worsen disease severity through neuromuscular or immune mechanisms (Županić et al., 2021; Cavalcante et al., 2013). Limited studies suggest that antiviral drugs (e.g., peramivir, oseltamivir, and the combination of interferon-alpha and ribavirin) may exacerbate or induce MG through neuromuscular or immune mechanisms, with evidence for oseltamivir currently limited to animal studies (Level 5) (Bektas et al., 2007; Fukushima et al., 2015; Hayashi et al., 2015). Pharmacokinetic interactions between antivirals and ISs predominantly arise from CYP450 modulation, exemplified by nirmatrelvir and ritonavir (NMV/r)-induced CYP3A4 inhibition that markedly elevates tacrolimus [TAC]/CsA levels, necessitating protocolized CNI dose adjustments (TAC withheld 12 h pre-NMV/r; CsA reduced to 20% with therapeutic drug monitoring) (Level 5) (Dewey et al., 2023; Giguère et al., 2023; Devresse et al., 2022). The additive risk of adverse effects resulting from concomitant use cannot be overlooked. Coadministration of AZA and ribavirin is contraindicated due to synergistic myelotoxicity risks (Level 5) (Sparkes et al., 2019). Other risks include enhanced myelosuppression with ganciclovir/valganciclovir and mycophenolate and nephrotoxicity from cidofovir and TAC, among others (Level 4–5) (Kotton et al., 2024; Rerolle et al., 2007). Risk mitigation requires vigilant drug monitoring, dose titration, selection of safer alternatives, and multidisciplinary oversight, as detailed in Table 1.

A 300-patient MG cohort study revealed elevated latent tuberculosis (TB) infection incidence, particularly among elderly individuals, who demonstrated significantly increased risk (OR = 1.91, 95% CI 1.18–3.09) (Chen et al., 2023). First-line anti-TB agents, including isoniazid, rifamycins, pyrazinamide, and ethambutol, may precipitate MG exacerbations through two mechanisms: direct neuromuscular toxicity and pharmacokinetic interactions (Level 5) (Kuang et al., 2022). Current evidence is limited to isolated case reports, which describe MG flares and myasthenic crises following the initiation of these drugs, with symptom onset occurring within weeks and positive rechallenge evidence (Litovsky et al., 2021). Rifampicin, a potent CYP450 inducer, may reduce pyridostigmine bioavailability or IS efficacy (Level 5) (Asaumi et al., 2018), worsening MG control. As shown in Table 1, rifampicin exhibits multiple Grade D pharmacokinetic interactions (defined as necessitating therapy modification or dose adjustment) with essential MG ISs, including TAC, CsA, MMF, and GCs such as methylprednisolone and dexamethasone (Level 5) (Sparkes et al., 2019). Prednisone has relatively few interaction risks and may be the preferred option for corticosteroids in MG patients with concurrent TB infection. Comparative analyses in living donor liver transplant recipients revealed that rifabutin, a rifamycin derivative with attenuated CYP450 induction capacity, maintains antituberculosis efficacy while exhibiting substantially reduced drug‒drug interaction (DDI) potential compared with rifampicin (Level 3) (Wang et al., 2021).

### 7.5 Conclusion

Clinicians must maintain heightened vigilance toward antibiotic classes with documented neuromuscular blocking effects, the potential for inducing myasthenic exacerbations, and significant drug interaction risks with MG therapies. These iatrogenic risks synergistically impair neuromuscular transmission, predisposing patients to life-threatening clinical deterioration.

1. This table delineates the clinically significant pharmacodynamic and pharmacokinetic interactions that occur among three key therapeutic classes (glucocorticoids, conventional immunosuppressants, and calcineurin inhibitors) and various antimicrobial agents. It is organized by these three immunosuppressant categories and provides detailed risk classifications, mechanistic summaries, and corresponding clinical monitoring and management guidance.
2. The symbol “a” denotes serum drug concentration. An upward arrow (↑) indicates an increase, and a downward arrow (↓) a decrease, in serum concentration or therapeutic efficacy. DXM: dexamethasone; MP: methylprednisolone; NIH: National Institutes of Health; AST: American Society of Transplantation.
3. Risk Rating Definition: This classification system assesses the clinical severity of drug interactions and the supporting evidence level. Ratings are defined as follows: A (No known interaction); B (No action needed); C (Monitor therapy); D (Consider therapy modification). This aids clinicians in evaluating the risk-benefit ratio and determining appropriate management.

## 8 Administration of immunosuppressive drugs

### 8.1 Balancing immunosuppression and infection risk

While preceding sections have addressed general principles of antibiotic selection and their potential interactions with immunosuppressive agents, this section provides a dedicated and systematic examination of the risk-benefit calculus in modifying established immunosuppressive regimens during active CAP episodes. Specifically, we synthesize evidence to address three critical dimensions not previously explored in depth: the infection risks inherent to specific immunosuppressive drug classes in the context of CAP, evidence-based strategies for dose adjustment or temporary suspension of these therapies stratified by CAP severity and the management of disease relapse risk following immunosuppression modulation. The central focus is therefore not on drug interactions *per se*, but on the overarching clinical strategy for immunosuppression management during a concurrent respiratory infection.

Immunosuppressive therapy constitutes a cornerstone of MG management, yet its association with increased infection risk necessitates careful risk‒benefit evaluation. A population-based cohort study revealed a twofold increase in severe infection rates among MG patients receiving immunosuppression compared with matched controls (Level 2) (Kassardjian et al., 2020). Multivariate analyses identified PE, MMF use, and high-dose corticosteroids as independent predictors of infectious complications (Prior et al., 2018). Infections associated with immunosuppressive therapies are not pathognomonic of MG. Critical findings from a worldwide CAP investigation demonstrated that prolonged steroid administration, a main therapeutic approach for MG, constituted 45% of significant infection risk factors (Level 3) (Di Pasquale et al., 2019). This evidence underscores the quintessential therapeutic challenge in MG: achieving optimal disease control through immunosuppression while minimizing its inherent infection risk. Current clinical strategies, particularly regarding corticosteroid dosing paradigms, remain inadequately supported by high-level evidence.

### 8.2 Management of corticosteroids in MG patients with CAP

A retrospective study of 125 MG patients who achieved steroid-induced remission identified accelerated steroid tapering (<11.5 months) as a strong predictor of relapse (hazard ratio [HR] = 27.80), with bulbar-onset disease independently predicting postwithdrawal recurrence (adjusted HR = 3.59). These findings advocate prolonged tapering protocols and sustained immunosuppression for bulbar-involved patients (Level 3) (Su et al., 2022b). In a cohort of 93 MG patients with COVID-19, unsatisfactory conditions with lower forced vital capacity (FVC) and previous long‐term GC treatment, especially at higher doses, advanced age, the presence of cancer, and recent rituximab treatment, were identified as the most important predictors of severe COVID‐19 infection. This study specifically cautions against GC escalation during COVID-19-related MG exacerbations and recommends IVIg as the preferred rescue therapy (Level 3) (Jakubíková et al., 2021). High-dose GC initiation in MG carries a 50% risk of transient symptom exacerbation, necessitating restrictions to hospitalized patients receiving PE/IVIg for MC management (Level 3) (Narayanaswami et al., 2021).

Dose-dependent infection risks are further evidenced by rheumatoid arthritis studies showing adjusted relative risks escalating from 1.10 (<5 mg/day prednisolone) to 1.85 (>20 mg/day) (Level 3) (Dixon et al., 2011). Prednisone doses exceeding 10 mg/day are similarly correlated with elevated serious infection rates (Level 2) (Mena-Vázquez et al., 2024). However, current evidence-based guidelines strongly recommend the adjunctive use of GCs in hospitalized patients with severe CAP (Level 5). Owing to insufficient evidence, no specific recommendations can be provided for dosage and treatment duration (Chaudhuri et al., 2024). A meta-analysis revealed that hydrocortisone, but not other corticosteroids, was associated with reduced mortality and improved outcomes in severe CAP patients (Level 1) (See et al., 2024). In summary, GC management in MG-CAP requires stepwise tapering to prevent relapse, judicious high-dose GC use limited to hospitalized settings, preferential hydrocortisone selection in severe CAP, early IVIg/PE adoption for high-risk patients, and multiparameter monitoring (respiratory function, infection biomarkers, MG symptoms) to harmonize neuromuscular and infectious outcomes.

### 8.3 Considerations for non-corticosteroid immunosuppressive agents

Maintenance therapy for MG remission typically involves gradual corticosteroid tapering combined with steroid-sparing IS, such as AZA, CNI, MMF, and occasionally CTX (Level 5) (Narayanaswami et al., 2021). There is a paucity of systematic studies addressing IS modification in MG patients with respiratory infections, while existing research predominantly focuses on treatment cessation due to drug intolerance or adverse events (Lascano and Lalive, 2021). Prospective cohort data reveal the distinct toxicity profile of azathioprine: abnormal liver function occurs in 23% of users, driving a significantly higher discontinuation rate than that of MMF and MTX (Level 2) (Dodd et al., 2024). In contrast, TAC has comparable MG efficacy to that of cyclosporine but superior safety, exhibiting minimal hepatotoxicity and reduced nephrotoxicity (Fan et al., 2023). Several factors can affect relapse risk in MG patients following IS withdrawal, such as the rate of dose reduction, duration of therapy, combination treatments, and patient-specific characteristics, including disease severity and antibody titers (Level 3–4) (Zhang et al., 2020; Sanders et al., 2006). A Rapid TAC reduction ≥0.76 mg/year elevates relapse odds 5.66-fold (Level 4) (Bi et al., 2022). Notably, GC-TAC combination therapy achieves superior relapse prevention and therapeutic durability in generalized MG (Level 3) (Zhang et al., 2020).

### 8.4 Risk-stratified adjustment of immunosuppression during infection

Multiple studies investigating COVID-19 outcomes in MG patients have demonstrated that conventional ISs (AZA, MMF, CsA, and TAC) have no statistically significant impact on COVID-19 progression, indicating that these agents have not been proven to increase complication risks, alter the disease course, or adversely affect clinical outcomes in COVID-19 patients with MG (Level 3–4) (Jakubíková et al., 2021; Camelo-Filho et al., 2020). This observation aligns with findings from smaller cohorts demonstrating favorable outcomes in patients maintained on low-dose prednisone combined with immunosuppressive regimens (Level 4) (Anand et al., 2020; Ramaswamy and Govindarajan, 2020). Clinical studies indicate that TAC may exert protective effects against SARS-CoV-2 infection by suppressing hyperactive immune responses that mediate inflammatory cytokine storms and clinical deterioration and the use of TAC was associated with a better survival thus encouraging clinicians to keep TAC at the usual dose (Level 2) (Belli et al., 2021; Li et al., 2020). However, among the 11 MG patients diagnosed with or suspected of having COVID-19, three required ventilator support, and two elderly patients died due to COVID-19-related respiratory failure. This underscores the importance of close monitoring and proactive management during acute viral infections (Level 3) (Businaro et al., 2021).

Current practices of immunosuppression management during infections exhibit significant variability between US and European transplant centers. While immunosuppressive dose tapering during active infections remains a common yet evidence-limited practice, few rigorous studies have investigated protocolized adjustment of immunomodulatory regimens and clinical management in solid-organ transplant recipients, which predominantly relies on empirical experience (Shepshelovich et al., 2019; Roberts and Fishman, 2021) A COVID-19-era systematic review of kidney transplant recipients revealed disease severity-stratified approaches (Level 1): watchful waiting predominated in asymptomatic/mild cases *versus* aggressive modification in symptomatic patients, including antimetabolite suspension (75.3%). CNI management strategies differ significantly by disease severity: maintenance (48.4%) or dose reduction (19.7%) in mild cases *versus* complete withdrawal (31.9%) in severe infections. Graft function preservation was observed in 76.2% of the recipients, with parallel stability in renal function (76.17%) throughout the observation period (Angelico et al., 2021). Complete IS withdrawal, although reserved for ventilator-dependent critically ill patients, was correlated with increased sepsis-related mortality (OR 2.11, p < 0.01) (Level 3) (Kim et al., 2024).

Consequently, CAP severity may constitute the primary determinant for immunosuppression discontinuation in MG patients. Those receiving immunomodulatory therapies require rigorous infection surveillance, particularly given that lymphopenia (OR 0.88, 95% CI 0.75–0.96) and hyperglobulinemia (OR 1.16, 95% CI 1.02–1.35) independently predict ventilatory requirements (p < 0.05) (Level 4) (Su et al., 2022a). These hematological parameters demonstrate prognostic utility for CAP severity stratification, guiding personalized therapeutic strategies.

### 8.5 Conclusion and future perspectives

**FIGURE 1:**
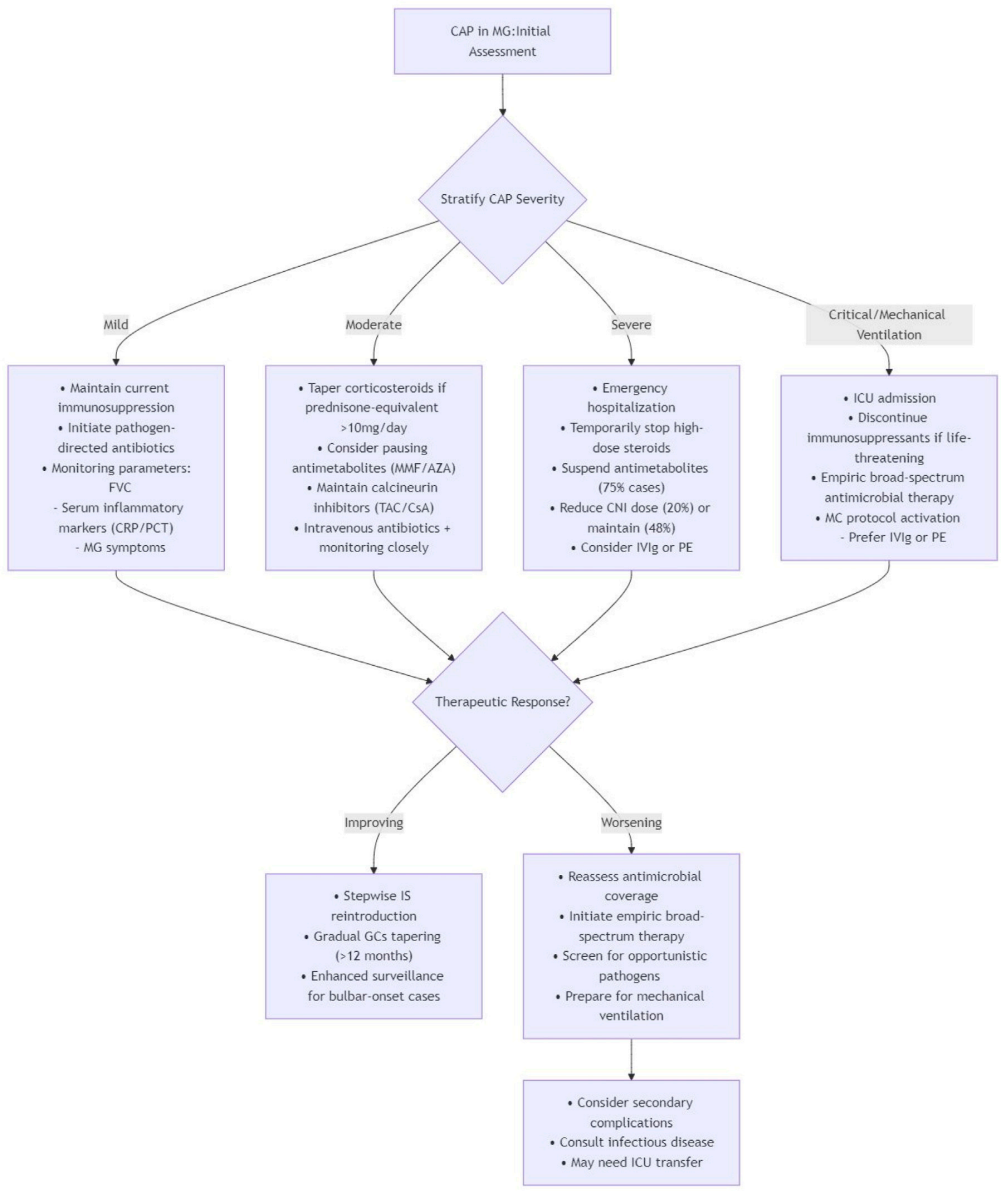
A risk-stratified approach for MG patients with CAP (Gilhus et al., 2019; Su et al., 2022a; Su et al., 2022b; Jakubíková et al., 2021; Dixon et al., 2011; Mena-Vázquez et al., 2024; Chaudhuri et al., 2024; See et al., 2024; Lascano and Lalive, 2021; Dodd et al., 2024; Fan et al., 2023; Zhang et al., 2020; Sanders et al., 2006; Bi et al., 2022; Camelo-Filho et al., 2020; Anand et al., 2020; Ramaswamy and Govindarajan, 2020; Belli et al., 2021; Li et al., 2020; Businaro et al., 2021; Shepshelovich et al., 2019; Roberts and Fishman, 2021; Angelico et al., 2021; Kim et al., 2024).

By integrating cumulative evidence, the management of immunosuppression in MG patients with CAP should adopt a risk-stratified approach to balance disease control against infection-related mortality (Figure 1). The development of precision immunosuppressive regimens, incorporating clinical variables such as age, MG phenotype (ocular/generalized), autoantibody profile (AChR/MuSK/LRP4), and comorbidity burden, warrants rigorous investigation, particularly regarding optimization strategies during concurrent respiratory infections. Multidisciplinary care coordination coupled with serial immunological monitoring has emerged as a crucial strategy for maintaining the delicate equilibrium between therapeutic immunosuppression and infection prevention.

To complement the risk-stratified management algorithm in Figure 1, we provide a Supplementary Table S3 offering practical, evidence-based guidance on key clinical dilemmas in managing immunosuppression for MG patients with CAP. It addresses common questions regarding drug prioritization for reduction, the role of bridging therapies, and high-risk patient identification, with corresponding recommendations, supporting evidence, and reference levels.

Tailoring immunosuppressive strategies according to CAP severity grading facilitates optimal management of both MG and infection control. This algorithm provides a structured framework for the initial assessment and management of MG patients with CAP, based on disease severity stratification. Key components include.

1. Initial assessment: Foundation based on clinical and diagnostic evaluation to stratify CAP severity into Mild, Moderate, Severe, and Critical/Mechanical Ventilation categories. 2. Severity-Specific Management:
  1. *Mild CAP:* Maintain current immunosuppression with pathogen-directed antibiotics and multiparameter monitoring (FVC, inflammatory markers, MG symptoms).
  2. *Moderate CAP:* Taper corticosteroids (>10 mg/day prednisone-equivalent), consider pausing antimetabolites, maintain calcineurin inhibitors, and initiate intravenous antibiotics with close monitoring.
  3. *Severe CAP:* Emergency hospitalization with temporary suspension of high-dose steroids and antimetabolites, reduction or maintenance of CNI dose, and consideration of IVIg/PE.
  4. *Critical CAP/Mechanical ventilation:* ICU admission with discontinuation of immunosuppressants if life-threatening, empiric broad-spectrum antimicrobial therapy, and preferential use of IVIg/PE for myasthenic crisis management.
2. Therapeutic response evaluation:
  1. *Improving:* Stepwise reintroduction of immunosuppressants with prolonged corticosteroid tapering (>12 months) and enhanced surveillance for bulbar-onset cases.
  2. *Worsening:* Reassessment of antimicrobial coverage, screening for opportunistic pathogens, preparation for mechanical ventilation, and consideration of infectious disease consultation or ICU transfer.

## 9 Vaccination for myasthenia gravis patients

*Streptococcus* pneumoniae and respiratory viruses such as influenza and SARS-CoV-2 are core respiratory pathogens for immunocompromised patients that can trigger or exacerbate MG (Prior et al., 2018; Aliberti et al., 2021). Consequently, vaccinations to protect against infections are an important part of the clinical management of these diseases. According to the literature, vaccines may induce similar immune cross-reactivity to what they are meant to prevent (Tayebi et al., 2023; Stübgen, 2010). However, no clear link between vaccination and myasthenia gravis has been demonstrated in the literature and no quantifiable excess risk of myasthenia gravis was identified following SARS-CoV-2 vaccination) (Level 5) (Willison et al., 2024).

### 9.1 Safety of SARS-CoV-2 vaccines

During the COVID-19 pandemic, study by assessed the safety of SARS-CoV-2 vaccines in a large cohort of MG patients, emphasizing the importance of vaccination given the high risk of severe COVID-19 in this population) (Level 4) (Farina et al., 2022). Similarly, an observational study investigated mRNA COVID-19 vaccines in patients with well-controlled MG, finding that vaccination was generally safe and well-tolerated in this group) (Level 3) (Gamez et al., 2022). A single-center case series study reported that the inactivated COVID-19 vaccines might be harmless in patients with MG with Myasthenia gravis Foundation of America (MGFA) score classification I to II, demonstrating the recommendation to promote vaccination for MG patients during the still expanding COVID-19 pandemic) (Level 4) (Ruan et al., 2021).

### 9.2 Safety of influenza and pneumococcal vaccines

Influenza vaccines, have been assessed for their safety in MG patients, and no association was found between the administration of influenza vaccines and the hospitalization of MG patients (Zhou et al., 2021; Auriel et al., 2011; Zinman et al., 2009) In 2017, a Korean study used a recall-based self-report questionnaire to demonstrate that the risk of MG symptom exacerbation following seasonal influenza vaccination was very low (1.5%), while the occurrence of influenza-like illness (ILI) was significantly associated with exacerbation of MG symptoms (40%) (Level 3) (Seok et al., 2017). These studies did not include patients with severe or unstable MG; Data on pneumococcal vaccination in MG patients are notably scarce. MTX treatment at the time of vaccination and increasing age were identified as predictors of poor vaccination outcome in multiple logistic regression analysis (Level 3) (Rasmussen et al., 2020).

### 9.3 Risk-benefit assessment and unmet needs

Though previously mentioned research on vaccination in MG patients is scarce and mainly about influenza vaccines, nearly all the evidence supports vaccine-related worsening of MG is rare (Level 1–4) (Auriel et al., 2011; Zinman et al., 2009; Seok et al., 2017; Strijbos et al., 2019). Study evaluating the cause of death in Swedish MG patients reveals influenza/pneumonia is a striking contributor (Westerberg and Punga, 2020). A 10-year longitudinal study found a significant 48% reduction in mortality and a 27% reduction in hospital admissions after influenza vaccination in autoimmune and autoinflammatory diseases (AIID) patients (Glück and Müller-Ladner, 2008).

Studies and guidelines in rheumatic diseases indicate that most live vaccines are contraindicated in patients with IS, while inactivated vaccines generally exhibit a similar safety pattern in immunosuppressed and immunocompetent patients, although the immune response to vaccination can be impaired or even absent with regards to magnitude, breadth, and persistence (Level 5) (Bijl et al., 2012; Rubin et al., 2013). However, data on the efficacy of vaccines in MG remains scarce, antibody titre testing to monitor responses can be considered where appropriate (Level 5) (Winkelmann et al., 2022).

### 9.4 Clinical recommendations and guidelines

Thus, most MG specialists believe the benefits of vaccination outweigh any small risks in possible transient MG symptoms exacerbation and For patients on IST, inactivated vaccines are recommended (MGFA Vaccinations, 2025). Guidelines also recommend influenza and pneumococcal vaccine for AIID patients, which preferably be administered during quiescent disease If possible, vaccinations should be administered prior to immunosuppressive drugs, but necessary treatment should never be postponed (Level 5) (Furer et al., 2020). Meanwhile, Alexander emphasizes that vaccinations must be avoided during relapses or exacerbations of neuroimmunological diseases (Level 5) (Winkelmann et al., 2022).

## 10 Conclusion

This systematic review advances the current understanding by comprehensively evaluating antimicrobial strategies for CAP in MG patients, encompassing evidence-based selection from empirical regimens to targeted therapies against multidrug-resistant pathogens, fungal coinfections, and viral complications. Clinicians managing CAP in MG patients must address three critical considerations when selecting antimicrobial agents: prevention of neuromuscular exacerbations, avoidance of clinically significant drug interactions with immunosuppressive therapies, and minimization of overlapping toxicity profiles. Effective clinical management requires proactive surveillance for adverse drug events, rigorous therapeutic drug monitoring, and individualized dose optimization on the basis of pharmacokinetic/pharmacodynamic principles.

Concurrent immunomodulator adjustment, guided by infection severity and evidence-based risk stratification, is essential to balance disease control with infection-related mortality risk. Through a systematic synthesis of extant evidence, we propose a risk-stratified algorithm for immunosuppressive management in MG patients with CAP. This decision-support framework addresses a critical evidence‒practice gap, providing interim guidance until formal consensus guidelines are established. Future implementation requires multidisciplinary collaboration, integrating neurology, pulmonology, and infectious disease expertise to optimize therapeutic outcomes.

Furthermore, this review underscores the critical role of preventive strategies, particularly vaccination against influenza, SARS-CoV-2, and *Streptococcus* pneumoniae. Proactive immunization significantly mitigates infection risk in this vulnerable population, forming an essential pillar of comprehensive MG management alongside antimicrobial therapy and immunomodulator adjustment.

Several limitations inherent in this work must be acknowledged. Although a comprehensive literature review was conducted, the generalized and heterogeneous nature of the available evidence constrained the granularity of our analysis. Crucially, we could not adequately account for critical variables such as MG autoantibody subtype (AChR, MuSK, LRP4), patient age, and specific immunosuppressive regimens-all of which are known to influence infection risks and therapeutic responses (Gilhus et al., 2018; Nelke et al., 2022). As a result, our findings and recommendations remain necessarily broad. To address these gaps, future research should prioritize large-scale, prospective studies specifically designed to delineate how these factors modulate infection risk and treatment outcomes. Such efforts are essential to establishing personalized, evidence-based management strategies for this vulnerable patient population.

## Glossary

MG: Myasthenia gravis
CAP: Community-acquired pneumonia
MC: Myasthenic crisis
IS: Immunosuppressant
NMJ: Neuromuscular junction
AchR: acetylcholine receptor
MuSK: Muscle-specific kinase
LRP4: lipoprotein receptor-related protein 4
GCs: Glucocorticoids
IVIg: Intravenous immunoglobulins
PE: Plasma exchange
ADRs: Adverse drug reactions
FcRn: Neonatal Fc receptor
AZA: Azathioprine
MMF: Mycophenolate Mofetil
US: United States
AST: American Society of Transplantation
MP: Methylprednisolone
MTX: Methotrexate
HR: Hazard ratio
CI: Confidence interval
NIH: National Institutes of Health
CNI: Calcineurin Inhibitor
NMV/r: Nirmatrelvir and Ritonavir
CsA: Cyclosporine
FVC: Forced vital capacity
CTX: Cyclophosphamide
SMZ-TMP: Sulfamethoxazole and Trimethoprim
CYP450: Cytochrome P450
TAC: Tacrolimus
DDI: Drug‒Drug interaction
TB: Tuberculosis
DXM: Dexamethasone
NIH: National Institutes of Health
MGFA: Myasthenia gravis Foundation of America
ILI: Influenza-like illness
AIID: Autoimmune and autoinflammatory diseases
